# Online tutorial on survival analysis for biomarker discovery

**DOI:** 10.1371/journal.pcbi.1014046

**Published:** 2026-03-20

**Authors:** Jaka Kokošar, Ela Praznik, Martin Špendl, Nancy P. Moreno, Alana Newell, Gad Shaulsky, Blaž Zupan

**Affiliations:** 1 Faculty of Computer and Information Science, University of Ljubljana, Ljubljana, Slovenia; 2 Department of Education, Innovation and Technology, Baylor College of Medicine, Houston, Texas, United States of America; 3 Department of Molecular and Human Genetics, Baylor College of Medicine, Houston, Texas, United States of America; Montreal, CANADA

## Abstract

In biomedicine, survival analysis addresses time-to-event data to study outcomes like patient survival and treatment response, and supports biomarker discovery. Yet, teaching this analysis is often hindered by mathematical and programming barriers. We present a structured, hands-on tutorial that goes beyond a typical online guide—offering integrated video lectures, literature, quizzes, and practical exercises. Built around Orange Data Mining, an open and free no-code visual analytics platform, the tutorial covers key concepts such as censoring, Kaplan-Meier curves, group comparisons, and biomarker discovery through real-world datasets. Organized in four pedagogical units, it progresses from basic survival data analysis to gene and gene-set biomarker discovery. Designed for 2–3 hours of learning, it supports both individual study and classroom use, and was successfully tested with over 120 participants.

## Author summary

In teaching data science, one of the biggest challenges is engaging the audience. Hands-on learning is a powerful way to do this—after all, the field itself starts with data. Another key consideration is the choice of tools: for true accessibility, the lesson should flow smoothly, keeping the focus on core concepts rather than technical hurdles. This is especially important in introductory courses for learners coming from application domains such as biomedicine. With this in mind, we’ve created a straightforward, hands-on tutorial for survival analysis and biomarker discovery, designed for both self-learners and educators. The tutorial is short, intuitive, and includes videos, written notes, quizzes, and practical exercises. All materials—including the tutorial, datasets, and software—are free and ready to use.

## Introduction

Survival analysis is a collection of statistical methods tailored to studying the time until an event occurs, such as disease progression, response to treatment, or patient mortality [1]. Its applications span from clinical trials to biomarker discovery, and the field’s relevance is reflected in the thousands of biomedical studies that rely on these methods each year [2]. However, survival analysis asks a different kind of question than many familiar statistical methods: rather than summarizing outcomes at a single time point, it follows individuals over time [3]. Consequently, these methods must account for ’censored’ subjects—those who do not experience the event during the study period. This presents a unique conceptual challenge: distinguishing informative censoring from missing data is critical, yet often non-intuitive for beginners. Furthermore, interpreting the resulting visualizations, most notably Kaplan-Meier curves, requires specific visual literacy. The distinctive ’steps’ of the curve and the diminishing number of subjects at risk over time differ significantly from standard statistical plots. These nuances are highly prone to over-interpretation and require careful introduction, as noted by Pocock et al. [4]. Given these complexities, it is crucial that students and researchers working with patient data gain an early and intuitive understanding of survival analysis.

As we show in our report, survival analysis can be introduced in a way that is accessible and engaging. By combining visual analytics with structured, real-world examples, we can teach key concepts without overwhelming learners with formal mathematical detail. With the right tools, learners can formulate hypotheses, visualize results, and reflect on the biological significance of their findings.

In this paper, we present a four-chapter online tutorial that covers the basics of survival analysis and demonstrates how to apply these concepts to biomarker discovery. The tutorial includes self-paced lecture notes, consisting of video and written materials, as well as hands-on exercises. These exercises guide learners through data creation, the design of analysis workflows, the interpretation of survival visualizations, and biomarker discovery tasks. The tutorial is specifically designed for biomedical students, regardless of their prior experience with computational analysis.

Our goal was to create a straightforward tutorial with a gentle learning curve, allowing the material to be covered in two to three hours. The tutorial can be used for independent study with self-assessment or as a resource for educators, providing both structured content and practical exercises for integrating survival analysis into a short, one-day course. Both the training materials and the accompanying software, Orange Data Mining, are open-source and freely available for classroom adoption.

### Didactic approach

The online tutorial takes a learner-focused approach to teaching survival analysis, prioritizing accessibility and active engagement. Recognizing that many biomedical students may not be proficient in computational programming, we employ a visual analytics framework that allows users to interactively explore survival data using no-code approach. The tutorial uses visual programming to assemble computational units and data visualizations into a concise analysis pipeline, enabling learners to interact with visualizations using techniques such as brushing and linking. This approach supports on-the-fly explorative data analysis, a much-needed approach in data science training [5]. The proposed tutorial provides an intuitive learning experience, where concepts such as censoring, Kaplan-Meier estimators, and biomarker discovery are introduced progressively through interactive workflows. This approach ensures that learners can focus on understanding survival analysis principles rather than being hindered by programming complexity.

The instructional design follows a structured progression, beginning with fundamental survival analysis concepts and continues toward biomarker discovery. Each unit builds upon the previous one, reinforcing key ideas through a combination of self-paced lecture notes, video tutorials, and hands-on exercises. The tutorial integrates real-world biomedical datasets, allowing students to work with authentic data and interpret survival curves in a meaningful context. Through guided problem-solving activities, learners apply statistical methods to compare survival outcomes across different patient groups, providing a deeper understanding of the field’s practical applications.

Active learning is at the core of this tutorial. Rather than passively absorbing information, students actively engage with data through exploration, hypothesis formulation, and interpretation of results. The tutorial promotes experimentation, by allowing learners to adjust datasets, explore various stratification approaches, and critically evaluate survival predictors. Self-assessment components, that includes quizzes and practical workflow exercises, reinforce understanding and develop practical skills. This combined approach ensures students build both theoretical knowledge and practical expertise in survival analysis, effectively preparing them for future research or clinical practice.

### Software and tools

The online tutorial utilizes Orange Data Mining [6,7], a free, open-source data science toolbox that supports interpretable workflows [8], interactive visualizations, and visual analytics. While Orange is a general-purpose machine learning and data visualization tool, the survival analysis functionality covered in this tutorial is provided through an additional open-source add-on.

There are a number of excellent tools, including those with web interfaces, that address survival analysis. Examples include KMPlotter, [9] cBioPortal [10] and others [11], which can support rapid validation of biomarkers and include data from large public data repositories. These tools, however, in general restrict analysis to pre-loaded datasets and fixed pipelines, and were not designed for training where the inspection of results of intermediate processing steps could provide valuable insight for the trainees. Such under-the-cover insight could be provided in scripting environments, such as those employing R and Python, but would require programming expertise from trainees, and result in longer training time otherwise reduced by means of tools that use visual analytics [12].

Compared to fixed pipeline on one side and scripting tools on another side of the toolbox spectra, Orange provides a middle ground. Its visual programming environment makes the analytical workflow explicit, requiring users to logically connect data processing units while maintaining an intuitive, code-free interface. Similar visual programming tools, such as KNIME [13] and RapidMiner [14], offer comparable workflow-based approaches, however, their execution models typically require discrete configuration and execution steps contrasted with Orange’s interpreted workflows, where results and changes in visual depictions are instantly available upon any change of input data or parameters of any of the components. Although Orange is also a general-purpose data mining tool, it has been designed by educators with training in mind, including support to peeking into intermediate results and explaining the utility of individual components in the workflows [15].

The pedagogical units presented in this tutorial are concept-driven and could be adapted for teaching in R, Python, or other similar tools. The lecture notes, tutorial, and quizzes are packaged into a mini-MOOC-like website, which is freely accessible online.

### Pedagogical units

Our proposed training consists of four core units, progressing from basic survival analysis to advanced biomarker discovery. In the first unit, we introduce the concept of censoring, explain that survival data always includes two features (time to last observation and event occurrence), teach how to construct a survival dataset, and present the data using a Kaplan-Meier plot. The second unit demonstrates how subjects in survival analysis can be characterized by various features, enabling patient stratification into groups with different survival outcomes. We then extend these concepts to data in which the features are gene expression levels. In the third unit, we show how patients can be stratified based on the expression of specific genes, and in the fourth unit, based on the collective expression of genes within predefined gene sets.

### Unit 1: Survival data, censoring, and Kaplan-Meier estimator

**Fig 1:**
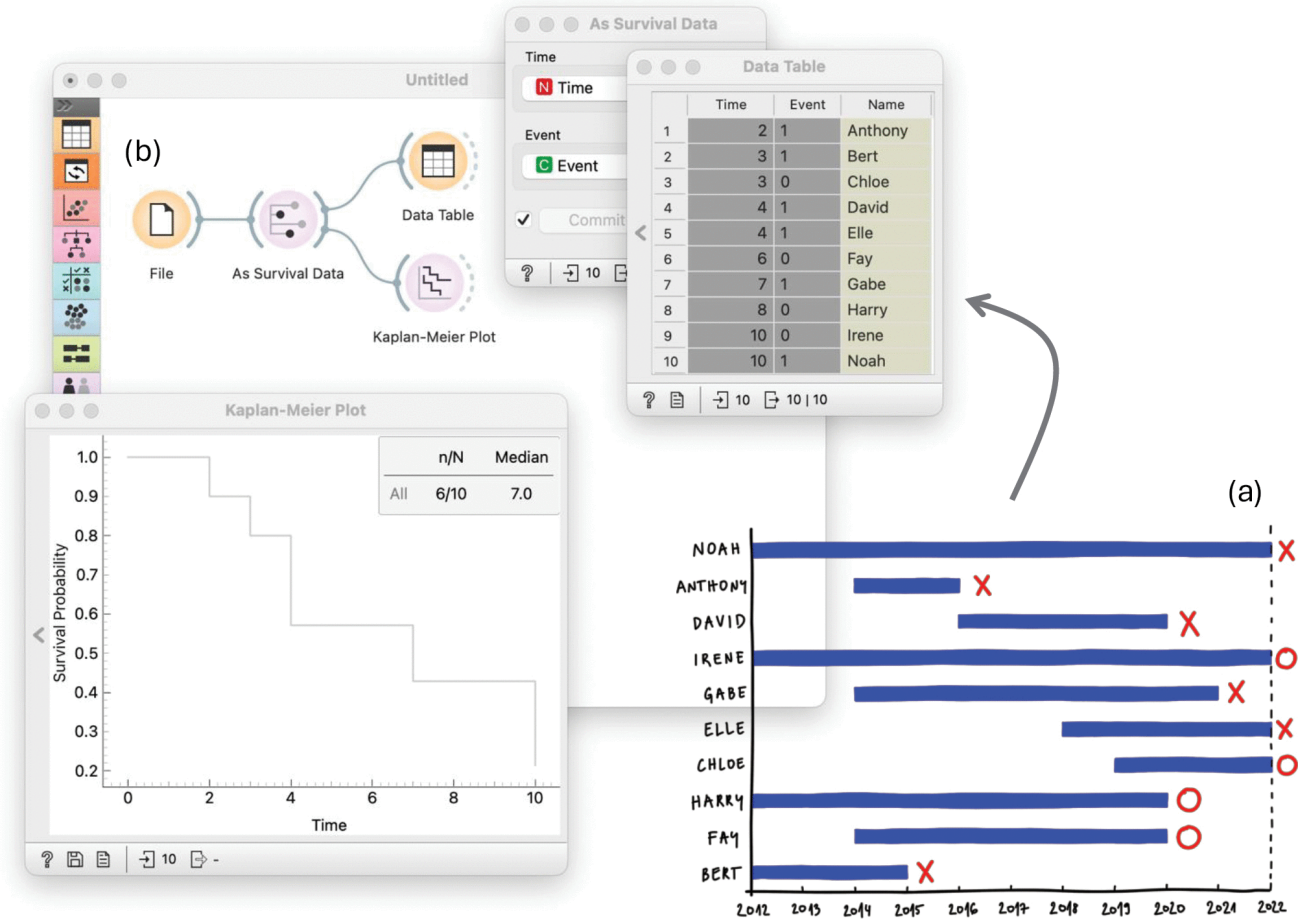
Orange workflow for Kaplan-Meier survival curve estimation. The workflow automates the Kaplan-Meier survival analysis using trainees’ prepared dental filling data. **(a)** Initially, data created from timeline diagrams is loaded into the workflow. **(b)** The *Kaplan-Meier plot* widget calculates and plots the survival curves, allowing trainees to visualize and confirm results obtained from manual analysis. Creating this workflow supports learners in developing an intuitive understanding of how censoring and time-to-event data are translated into stepwise survival curves.

A key challenge in survival analysis is handling censored data—cases where the event of interest has not yet occurred within the study period. In this unit, trainees learn to recognize censoring, create their own survival data from the description of the study, and plot survival curve using the Kaplan-Meier estimator [16]. The unit follows a step-by-step approach, combining conceptual explanations with hands-on data preparation and visualization:

1. Conceptually, as a story, introduce a relatable, illustrative example to introduce survival analysis concepts and the problem of censoring. The proposed case in our material involves a dental study that aims to understand the durability of dental fillings over 10 years.
2. Start with the data in the form of a timeline diagram (Fig 1A), which shows the time to failure after a dental filling procedure (marked with X) or censoring (marked with O).
3. Discuss censoring by distinguishing between observed events and censored cases. Introduce right censoring and explain why it must be handled properly rather than treated as missing data.
4. Convert the timeline diagram into a structured survival dataset with a time variable and an event indicator (1 for event occurrence, 0 for censoring).
5. Introduce the Kaplan-Meier estimator as a method for estimating survival probabilities over time. Explain the survival function, which represents the probability of surviving past a given time. Guide trainees through stepwise probability calculations using the dataset. Show how censored data points contribute to the analysis without artificially inflating survival probabilities.
6. Manually plot the Kaplan-Meier survival curve and explain that it is one of the most commonly used plots in survival analysis. Introduce the concept of median survival time as the time at which survival probability drops to 50%.
7. Prepare the data using spreadsheet software.
8. Build a data analysis workflow that automates this analysis (see Fig 1B). The workflow should import the data, show the data in a table, calculate the survival curve, and plot the results. At this stage, students should be able to reproduce the analysis they have done manually.

### Unit 2: Group comparisons

Observations in survival datasets are typically characterized by various features beyond time-to-event and censoring information. We can use these features to form groups of observed subjects (data instances) and then compare their survival curves. Features that lead to groups with distinct survival characteristics may be considered as important survival predictors. Finding such features and their combinations is one of the fundamental steps in survival analysis.

**Fig 2:**
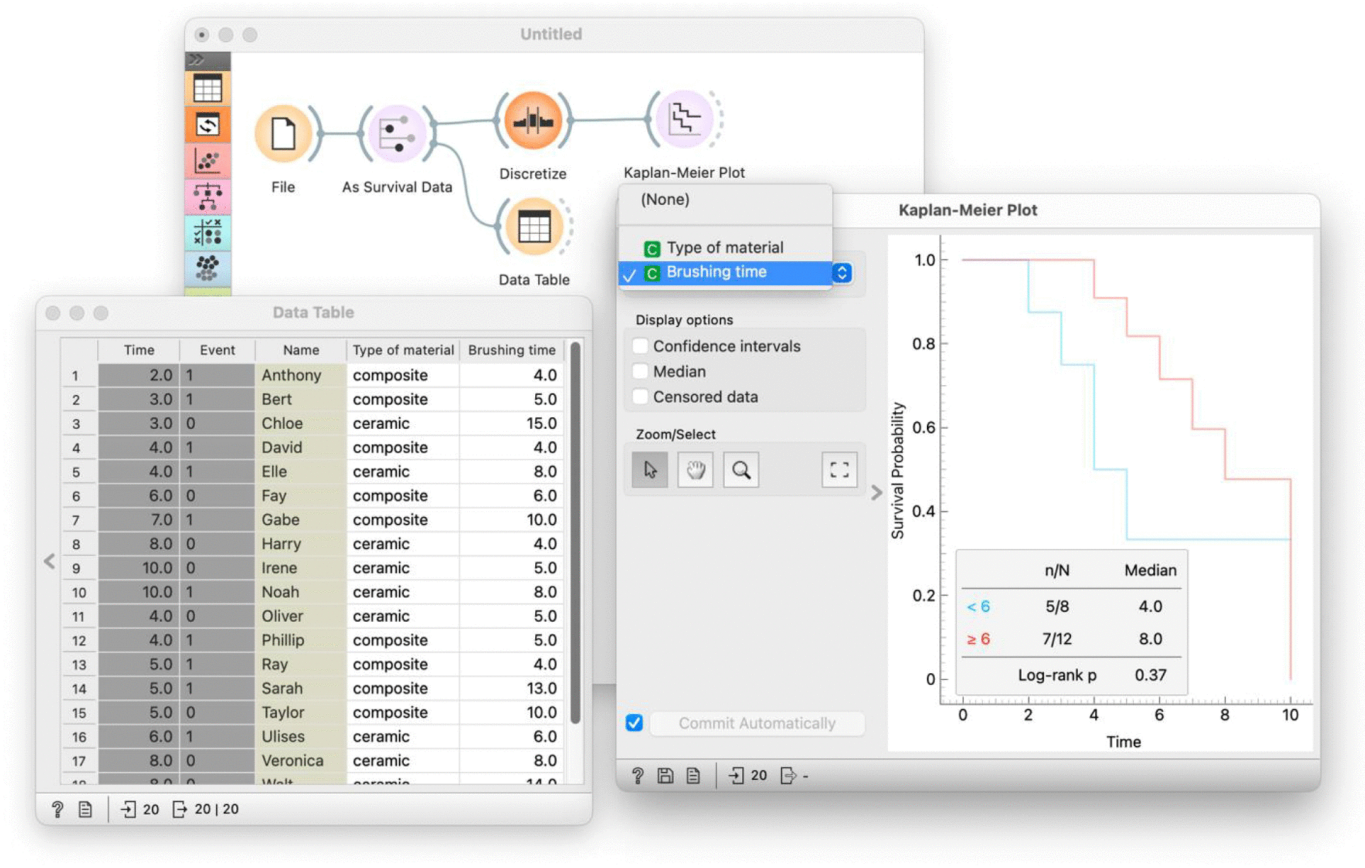
Orange workflow demonstrating stratification and comparison of survival curves. This workflow guides trainees through the grouping of data based on a continuous variable. The *Select Rows* widget partition data into groups (e.g., less than vs. greater than or equal to 6 minutes daily). *Kaplan-Meier plot* widget visualize survival curves and computes the log-rank test. Trainees can interactively select different variables to explore their impact on patient survival, and by using the workflow understand how feature-based stratification influences survival curves and statistical significance.

1. Expand the dental fillings dataset to include additional features, such as the type of material used in the filling and the average brushing time of each individual. Add these features to the spreadsheet file.
2. Identify categorical features that naturally define groups. For example, the feature “type of material” naturally split the data into two groups. Plot Kaplan-Meier curves for composite versus ceramic fillings and interpret the differences between them.
3. Forming groups based on continuous features requires defining a meaningful threshold. Use brushing time as an example and create groups of individuals who brush more or less than 6 min daily (Fig 2). Observe the results of such stratification in the Kaplan-Meier plot.
4. Compare the results between the two groupings. Point out that, intuitively, the separation of the survival curves is more pronounced for material type compared to brushing time.
5. Introduce the log-rank test [17] as a statistical method to assess the significance of survival differences between groups, and observe the differences in the *p*-values between the two groupings.
6. It is now time to consider a more complex dataset. We propose to dive into the German Breast Cancer Study Group (GBSG) dataset [18], which contains survival data for breast cancer patients. The dataset includes various clinical and pathological features, such as tumor size, lymph node status, and hormone receptor status. The goal is to identify features that significantly impact patient survival based on the log-rank test, and inspect which features produce the most significant splits in the survival curves.

### Unit 3: Finding gene markers

Identifying biologically meaningful genes that impact survival is an important step in biomarker discovery. In this unit, trainees learn to stratify clinical cohorts based on gene expression values, and assess their association with survival outcomes. The workflow begins by evaluating a known prognostic gene (*KRAS*), demonstrating how to partition patients into high- and low-expression groups [19]. The focus then shifts to data-driven hypothesis generation, where trainees apply feature ranking to explore larger collections of genes and prioritize those with the largest correlation with survival. By systematically analyzing genes within a specific biological pathway, trainees also learn to uncover and prioritize markers with potentially stronger predictive value than those initially examined based on related reports from the literature.

1. Introduce a feature-rich dataset, such as TCGA-BRCA [20], which contains gene expression data for breast cancer patients and includes tens of thousands of genes. Discuss the challenges of working with biomedical datasets, including the vast feature space, and the opportunities for personalized medicine [19,21]. Emphasize the biological importance of identifying genes whose expression levels significantly correlate with patient survival, aiding in cancer prognosis and treatment strategies.
2. Load the data and highlight the known prognostic gene *KRAS* as an example [22,23]. Visualise KRAS expression values in a distribution plot, decide on a threshold, and plot the resulting Kaplan-Meier survival curves for patients with high and low expression levels. How does overexpression of *KRAS* affect survival?
3. Use the log-rank test to assess whether the separation of survival curves by expression of *KRAS* is statistically significant.
4. *KRAS* gene is a member of the RAS signaling pathway [24]. Consider about 200 other genes from this pathway. Rank them based on their association with survival outcomes. Are there other genes that are more predictive of survival than *KRAS*? Trainees can find that the gene *FLT3* has a more significant association with survival than *KRAS*, but interpreting its effect is different: overexpression of *FLT3* leads to a more favorable prognosis, while overexpression of *KRAS* leads to a worse prognosis.

### Unit 4: Finding informative gene sets

Gene sets, collections of genes representing specific biological pathways or processes, offer insights beyond individual gene-level analyses and can help us better understand the biology of the underlying diseases [25].

**Fig 3:**
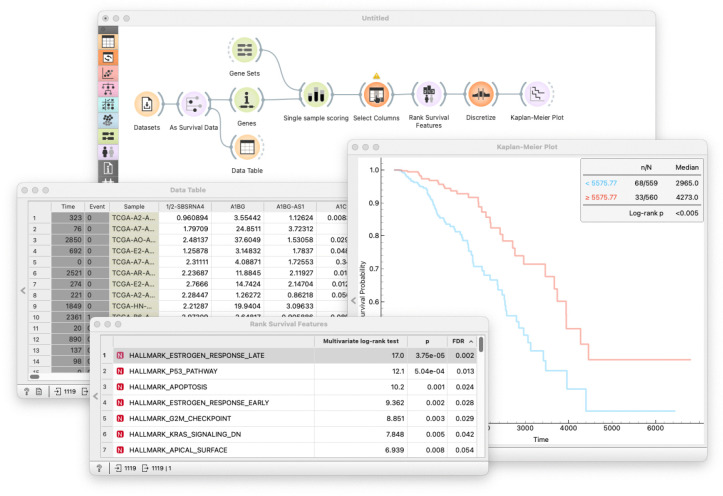
Gene set enrichment analysis workflow for survival prediction. The workflow guides trainees through the process of calculating ssGSEA enrichment scores for a specific gene set and stratifying patients based on these scores. The workflow stratifies patients based on ssGSEA scores and visualizes Kaplan-Meier survival curves, revealing the prognostic impact of biological pathways. Users can experiment interactively with different gene sets to identify biologically meaningful predictors of survival. We use this workflow to support learners in understanding how pathway-level features may provide more robust and interpretable survival predictors than individual genes.

1. Introduce single-sample gene set enrichment analysis (ssGSEA) [26] and explain how it can be used to represent the activity of a collection of genes as a single continuous feature.
2. Consider the TCGA-BRCA dataset. Select only genes that belong to a specific gene set. Use the hypoxia gene set as an example and calculate the ssGSEA enrichment scores for each sample. Hypoxia is closely related to cancer [27] because low oxygen levels in the tumor microenvironment can drive tumor progression, resistance to treatment, and poor patient outcomes.
3. Use the ssGSEA enrichment score to stratify the data. This step follows the same procedure as the previous pedagogical unit. To simplify the analysis, reduce the dataset to include only the survival features and the calculated enrichment scores. Display the distribution of the scores, define a threshold, and plot the Kaplan-Meier survival curves for patients with high and low enrichment scores. How does the overexpression of hypoxia-related genes affect survival? Are the results significant?
4. Experiment with different gene sets. For example, in our lecture notes, we propose using established Hallmark cancer gene sets [28]. Start with the hypoxia gene set and analyze its impact on survival.
5. Instead of selecting a specific gene set, consider all gene sets and rank them based on their association with survival outcomes (Fig 3). This introduces the concept of gene-set ranking, which is generally more robust than ranking individual genes. In this unit, trainees discover that some gene sets from the Hallmark collection are highly predictive of survival and have clear biomedical interpretations.

### Tutorial evaluation

**Fig 4:**
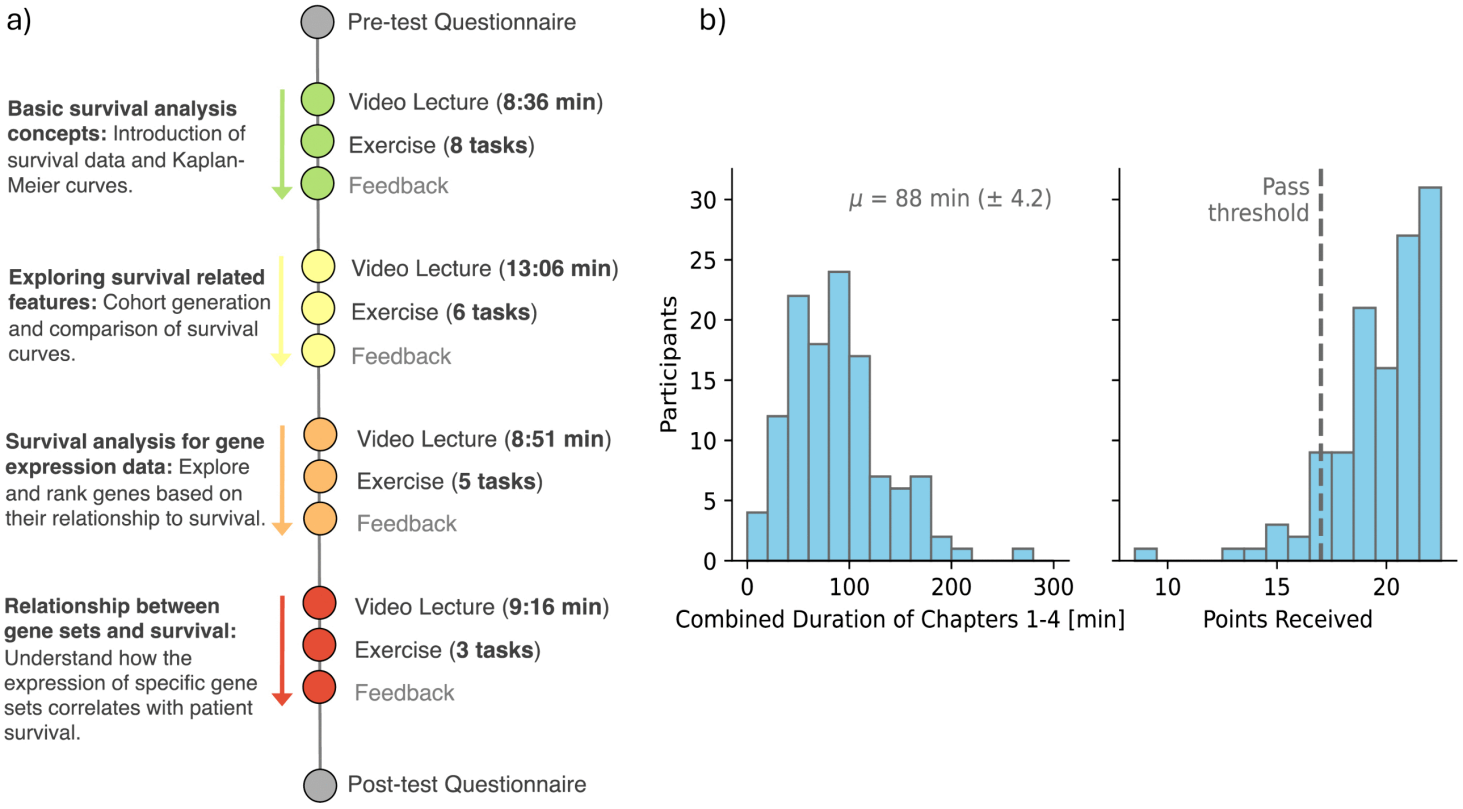
(a) Overview of the user study: a pre-test questionnaire, four content chapters, and a post-test. The tutorial includes approximately 40 min of video and 22 exercises, progressing from basic survival analysis to biomarker discovery. **(b)** Distribution of time spent and scores: participants spent an average of 88 min. With a 22-point maximum, 93% (113 participants) passed by scoring above 17.

We have piloted the tutorial with about 120 participants who were motivated to self-train in survival analysis. Through our communication channels, we engaged biomedical students at Baylor College of Medicine and University of Ljubljana, who had already completed the introductory data science course in Orange, as well as members of the broader Orange community, most of whom had limited to no background in survival analysis. Participants completed the four-part tutorial that aligns with the pedagogical units discussed above. Each chapter consisted of embedded video lectures, readings, and content knowledge tests aligned with the chapter objective. After each chapter, we asked the participants to evaluate the content and their learning experience. Fig 4A outlines the composition of the designed tutorial.

The study protocol was reviewed and approved by the Research Data Handling Board of the University of Ljubljana (Approval Number: 20230705001) and the Institutional Review Board of Baylor College of Medicine (Approval Number: H-53341). Participation in the tutorial was strictly voluntary. Participants were informed in writing via email and the tutorial landing page that by submitting their responses, they provided written informed consent to participate in the study. Users retained the right to withdraw at any time and could delete their stored answers. All data were anonymized prior to analysis, and no personally identifiable information was included in the reported results.

To better understand how participants engaged with the material, we monitored success rates and completion times. The average time spent on the exercises was about 88 minutes (see Fig 4B). Taking into account an additional 40 min of video lectures, the entire training took about two hours, which closely aligns with our tutorial design goals. Most participants were able to build and apply survival analysis workflows correctly, and quiz results showed a solid grasp of the material—over 90% of the participants scored above the passing threshold, which is encouraging for self-directed study.

The step-by-step structure, starting with basic concepts and moving toward biomarker discovery, seemed to support learning well. One participant noted, *“Although I don’t know much about bioinformatics, it was clearly explained,”* while another said, *“Very well-structured.”* Many found the hands-on approach helpful, with comments like *“Good exercise covering both mathematical concepts as well as a solution via Orange,”* and *“It has become easier to solve the problems that are mentioned in the quiz section.”* Several participants mentioned difficulty with biomedical terms—*“There are too many words I’ve never heard about”*—but this was often framed as a manageable challenge. Based on this feedback, we made minor changes to improve clarity. Overall, participants reported increased confidence in applying survival analysis on their own and appreciated learning through visual programming and real-world datasets. Many highlighted the value of being able to replicate published results and explore genomic data in a structured and guided format.

## Discussion

The proposed online tutorial is a structured and openly accessible introduction to survival analysis where we have emphasized interactive, problem-based learning. Hands-on exercises are coupled with intuitive explanations of key theoretical concepts. The tutorial enables learners to explore Kaplan-Meier survival curves, discover and describe groups with different survival characteristics, and identify potential biomarkers without requiring programming expertise. Our hands-on and problem-based approach lowers the barrier and smooths the learning curve for biomedical students and researchers and, by following the principles of visual analytics, provides a responsive training environment.

The presented hands-on training approach aligns with best practices in data science education, as it emphasizes both active learning and computational reproducibility. The tutorial employs active learning strategies that have been shown to significantly improve student performance and retention in STEM fields [29]. Furthermore, the use of visual programming enforces a structured analytical process and provides an alternative to ad-hoc spreadsheet manipulations, which may be error-prone and difficult to trace [30]. Saved visual workflows in Orange may also serve as a self-documenting record of the analysis. This implicitly introduces students to the concept of reproducible computation—a critical competency in modern biomedical research [31]—and ensures that their analytical path is transparent and reproducible.

Despite these advantages, there are some limitations. The tutorial focuses on fundamental survival analysis methods, and more advanced techniques such as Cox proportional hazards modeling and time-dependent covariates are not covered. Additionally, while the tutorial introduces biomarker discovery using survival analysis, the interpretation of biological findings requires domain expertise that extends beyond the scope of the training.

As such, the tutorial can serve both as an initial survey of statistical and machine learning methods in the field for those planning to explore further, and as a self-contained overview for users who seek familiarity with survival analysis.
